# Biodeterioration of Cement and Cement–Polymer Mortars: Analysis of the Influence of the Structure and Distribution of Pores on the Humidity of Mortars Exposed to the Biological Environment

**DOI:** 10.3390/ma17030612

**Published:** 2024-01-26

**Authors:** Elżbieta Stanaszek-Tomal

**Affiliations:** Faculty of Civil Engineering, PK Cracow University of Technology, 24 Warszawska Street, 31-155 Cracow, Poland; estanaszek-tomal@pk.edu.pl

**Keywords:** biodeterioration, absorbability, distribution of pores, porosity

## Abstract

The biodegradation of building materials refers to the problem of loss of performance due to biological agents, mainly dry rot fungi, moulds (filamentous fungi), bacteria and insects. Biocorrosion not only leads to the damage and deterioration of building materials, but can also pose a direct threat to human health. Inorganic building materials are a difficult substrate for microorganisms because they need food sources for their metabolism. However, they become colonised by microorganisms. In this paper, the effect of mould fungi on the moisture content and structure of CEM I and CEM I cement–polymer mortars with a 5% polysiloxane latex admixture was analysed. The analysis was carried out after 15 months of exposure to the biological environment. It was found that the cementitious materials were susceptible to the corrosive environment in the form of the filamentous fungi *Penicillium chrysogenum* and *Cladosporium herbarum*. It was also found that, after 15 months of exposure to mould fungi, CEM I cementitious materials without admixtures were slightly less susceptible to mould fungi than CEM I with the addition of a 5% polysiloxane admixture.

## 1. Introduction

Nowadays, the construction industry is required to use materials to achieve highly durable structures. The most important of these materials is concrete, which has become a basic building material. Concrete has many advantages, but it has a high porosity, which can contribute to accelerated chemical, physical and biological corrosion. The costs associated with repairing such structures are very high, which is why this material is subject to constant modification. The modification of this construction material is related to a change in composition, which can consequently affect the properties before and after the hardening process. The improvement depends on the type of cement, the w/c ratio and the use of organic and/or mineral admixtures and additives. Organic compounds include macromolecular compounds, i.e., polymers. Their introduction is intended to improve and strengthen the binder. It also allows additional spatial structures to be obtained. Such materials can be called cement–polymer composites [1].

Polymer–cement composites are superior to ordinary concrete or cement mortar in their properties. Such properties include their flexural and tensile strength, adhesion to various substrates and resistance to certain external agents. They can be divided into the following three groups on the basis of their composition and their production:

- Resin concrete (PC), formed during the cross-linking process of a two-component resin with selected fillers and aggregates;
- Cement–polymer concrete (PCC), formed during the curing process of a fresh cement mixture together with non-reactive polymers (pre-mix) or reactive monomers (post-mix);
- Polymer-impregnated concrete (PIC), which is produced by impregnating hardened concrete with a monomer or oligomer that polymerises and then cross-links the formed polymers within the pores of the concrete [2,3]. PCC (polymer–cement concrete) binders are obtained by adding a polymer, oligomer or monomer to the concrete mixture.

Based on the chemical reactivity of the modifier, the following systems are distinguished [4]:

- PCC which polymerises after mixing (post-mix), where chemically active, chemically curing synthetic resins or suitable monomers or prepolymers are introduced into the concrete mixture; their polymerisation occurs simultaneously with cement hydration;
- PCC polymerised before mixing (pre-mix), where practically chemically unreactive polymers are introduced into the concrete mixture; their modifying effect is primarily physical.

Two processes take place during the setting of -pre-mix PCC cement–polymer mixtures, i.e., cement hydration and the formation of a continuous polymer film (coalescence), due to the binding of water in the cement and its partial evaporation.

In the case of post-mix PCC, an additional reaction takes place between the resin and the amine hardener, resulting in the spatial cross-linking of the polymer. The hydration and coalescence processes are in competition with each other. Premature formation of the polymer film hinders or prevents the hydration of the cement. It is important to select the rate of these processes so that cement hydration precedes coalescence. As a result of the correct course of these processes, a polymer–cement microstructure with two interpenetrating structures is formed: polymer and cement [5]. When determining the *w*/*c* (water–cement ratio) of PCC mixtures, the water contained in the polymer dispersion should be included in the *w*/*c* value.

In addition to this, there are pores that are filled with a gas or a liquid. These can be open or closed pores. Their shape and volume depend primarily on the water–cement ratio, the mineral composition of the Portland clinker, the quality and quantity of mineral additives or chemical admixtures. The bulk of the pores may be filled with a liquid in the form of a liquid electrolyte containing calcium and sodium and potassium ions. This solution has a pH of over 13. Pores also occur in the so-called contact zone, formed at the interface between the aggregate and the hardened slurry. They are formed during the mixing of the concrete components and are partially removed during compaction [6,7]. The pore structure in cementitious materials includes air voids, capillary pores and gel pores [8]. The commonly used classification by pore size, introduced by the IUPAC (International Union of Pure and Applied Chemistry), distinguishes between three pore classes: micropores < 2 nm wide, mesopores 2–50 nm wide and macropores > 50 nm wide. The boundaries between the different classes are conventional and were adopted based on adsorption criteria. Micro- and mesopores are important in adsorption processes and determine the size of the internal surface area. Macropores contribute little, but in sorbents they act as transport pathways, allowing access to pores of smaller size [9]. A simplified division is adopted [10]. The following pores will then be present in the structure:

- Gel pores < 10 nm;
- Associated with crystalline products 10–100 nm;
- Capillary 100–1000 nm;
- Macropores > 1 μm [11].

Both water absorption and chemical corrosion resistance depend on pore size. 

The parameters that can be used to characterize the structure of porous materials are: specific surface area, bulk porosity, density and permeability.

An increase in humidity can be caused by corrosive agents [10]. This includes factors such as chemical and biological agents. Biological agents can cause corrosion induced by micro-organisms such as fungi or bacteria as well as algae and lichens. Biological corrosion has been defined as the degradation of materials due to the decomposition of living organisms and/or the products of their metabolic activity [10]. The destruction processes of materials, stimulated by the activity of organisms, are defined as biodeterioration. In the case of filamentous fungi or bacteria acting as corrosive agents on technical materials, two mechanisms play an important role, namely biological decomposition and corrosion induced by microorganisms. The type of mechanism depends on the composition of the technical material.

The biodeterioration of cement-based binder materials occurs due to different mechanisms. It depends on whether the contaminated material can be a food source or not. Accordingly, the following mechanisms are distinguished:

(a) Chemical assimilative biodeterioration occurs when the material is degraded for its nutritional value. Materials that undergo this mechanism are those that contain organic compounds in their composition, e.g., adhesives, wood, etc.
(b) Chemical dissimilatory biodeterioration occurs when the metabolites (products of the metabolic process) of microorganisms damage the material. This can occur both by the proliferation of microorganisms and by reacting chemically with it, causing chemical corrosion, the release of toxic metabolites into materials and pigmentation. Materials susceptible to this type of mechanism include metals, concrete, mortar bricks, glass and rocks.

Mineral building materials are subject to microbe-induced corrosion and encrustation. As a result of these mechanisms, materials crumble, fracture and break down due to metabolic products or the microorganism itself. This increases the porosity of the materials. In addition, some by-products of the microorganisms (e.g., sulphuric acid) can cause the dissolution of material components. This also has the effect of increasing the porosity and reducing the strength of mineral materials.

Very often, microbial growth occurs on the surface of materials. A change in colour or pitting may then occur. The growth of micro-organisms and the subsequent action of various mechanisms can lead to changes in material moisture content, porosity or strength.

Industrial buildings as well as single or multi-family dwellings are exposed to attack by micro-organisms, in particular, filamentous fungi. The right conditions are required, and these include temperature and increased relative humidity. The right combination of these can cause fungal contamination. To a lesser extent, parameters such as high concentrations of chloride ions, carbon dioxide and other salts, sulphates and small amounts of acids also affect the growth of microorganisms.

During their life cycle, moulds secrete metabolic products. These include volatile products, organic acids, and other chemical compounds such as water. When they colonise a technical material, they form a biofilm on their surface, which has the task of, among other things, storing water and protecting the microorganisms from the external environment. Water in the material also appears due to the process of metabolism, which is why materials become damp. Therefore, the aim of this article is to investigate how the action of filamentous fungi affects the porosity and pore distribution of a cement–polymer mortar modified with a 5% polysiloxane admixture and the moisture parameters of these materials. Polysiloxane latex is a modifier of cement–polymer materials used in the construction industry to repair reinforced concrete structures. Given that it can be subject to biological corrosion, it was therefore used in this study. The use of a 5% admixture in cement–polymer mortar can be surprising. The 5% polymer additive in relation to the weight of the cement is interesting, because on the one hand, according to [12], this amount can be an admixture, and on the other, it can be a polymer additive. As such, for the first case, the amount of admixture is insufficient to form a separate continuous phase in the hardening concrete or mortar. On the other hand, in the second case, a continuous film may be formed which guarantees the formation of a mixed cement–polymer binder, where the function of the polymer is to coexist with the cement as a binder. It was interesting in this case to see what would ‘win out’ and how one way or the other would affect the properties of the material contaminated with filamentous fungi. For this reason, this amount of admixture was chosen.

## 2. Materials and Methods

### 2.1. Materials

Table 1 shows the composition of the cement used. 

Quartz sand was used as a fine aggregate to prepare mortar samples [13].

The water–cement ratio for the tested mortars was 0.5. The composition of the cement–polymer mortars tested is presented in Table 2. 

The samples were formed into beams in the form of cuboids with dimensions of 20 × 20 × 160 mm. They were made of mortar, the composition of which is presented in Table 2. The finished samples were stored under foil for 24 h. After removing them from the mould, the bars matured under foil for another two days. In the next stage, they matured at a temperature of +20 °C ± 2 °C and a relative humidity of 60% ± 5% (PN-EN 12190:2000: Products and systems for the protection and repair of concrete structures—Test methods—Determination of the compressive strength of repair mortar) [14]. For the next 28 days, the samples were stored in laboratory conditions.

### 2.2. Corrosive Environment

To analyse the impact of microorganisms on cement materials, two types of filamentous fungi were used, i.e., Cladosporium, which is the most common and most common in the air, and Penicillium, a common fungus [15,16].

Modified malt extract Agar M-8927 (MEA) from Biochemistry was used as the growth medium. The growth medium was used for the isolation, detection and determination of filamentous fungi. It was brought to boiling point and then sterilised in an autoclave at 121 °C for 15 min. The final pH of the growth medium at 25 °C was 4.6. 

Pure cultures of *Penicillium chrysogenum* (LOCK 0531, streina F00680) and *Cladosporium herbarum* (LOCK 0490, streina E123) were imported from the collection of pure cultures of microorganisms (LOCK) from the Institute of Fermentation Technology and Microbiology in Łódź, Poland. Pure cultures of filamentous fungi were transferred into 10 mL of distilled water. Petri dishes were inoculated with the selected fungal culture (suspension). Inoculated cultures were incubated at 25 °C and 95% relative humidity for 5 days. In the next step, a clean and sterile swab was taken and placed into 10 mL of distilled water (suspension with a spore count of 1 × 10^6^ spores/mL). The obtained suspension was contaminated with previously prepared samples of cement–polymer composite. For convenience, we will use abbreviations of the names of fungi: Penicillium chrysogenum will be P.ch. and Cladosporium herbarum will be C.h.

### 2.3. Temperature and Humidity Parameters

The contaminated samples in the form of trabeculae were placed in a test chamber for biological tests. The conditions inside the chamber were: temperature 25 °C and relative humidity 95%. 

### 2.4. Experimental Time

Tests were conducted at 3, 6, 9, 12 and 15 months after contamination with filamentous fungi. In this article, only results after 15 months are presented. The results after 3, 6, 9 and 12 months are presented in publication [17] and for licensing reasons cannot be included in this manuscript.

Reference samples, i.e., materials not contaminated with filamentous fungi and a cement–polymer material without admixture applied, were used for comparison.

### 2.5. Tested Parameters

#### 2.5.1. Mass Moisture (µ_M_)

The mass moisture was determined in accordance with PN-85/B-04500 [18]. In the first step, the samples were weighed and in the next step they were dried at 105 °C to obtain constant weight. Mass moisture was determined from the formula:

u_m_ = (m_w_ − m_s_)·100%/m_s_ [%mass.] (1)

where: m_w_—wet sample mass [g]; m_s_—dry sample mass [g].

#### 2.5.2. Water Absorption (Absorbability)

The samples being tested for absorbability were dried to constant weight in a dryer at 105 °C. In the next stage, the elements were placed in water at 25 °C. When the weight difference between two measurements at 24 h intervals was not less than 0.5%, the test was terminated. This parameter was determined in accordance with the ASTM standard (ASTM C 642) [19]. The water absorption parameter was calculated using the formula:

n_m_ = (m_n_ − m_s_)·100%/m_s_ [%mass.] (2)

where: m_n_—sample mass of water saturated material [g]; m_s_—sample mass of dry material [g].

#### 2.5.3. Degree of Moisture Permeation

The degree of moisture penetration is expressed as a percentage. It is the ratio of the moisture mass of the material to the moisture in the fully saturated state. The degree of moisture permeability indicates the percentage of the available water pore volume that is filled with water during the test [15].

#### 2.5.4. Degree of Saturation with Water

The degree of water saturation, in [%], determines the ratio of volumetric water absorption (absorbability) to porosity.

#### 2.5.5. Porosimetry

Porosity was determined using a Quantachrome Poremaster Nova1000e mercury porosimeter. With this method, the porosity and pore size distribution can be determined in the ray range from 4 to 30,000 nm. The diagnostic method involves filling the pores of the material with mercury under increasing pressure. The procedure for this test is described in detail in [20,21]:

1. Two small samples 10 mm in diameter and 10–15 mm long were taken from the test pieces. Each sample weighed approximately 2 g;
2. The samples were dried in an oven at 105–110 °C for 24 h;
3. Samples were stored in a desiccator until testing.

The test results were presented in the form of graphs showing the dependence of pore volume on pore diameter. The results were processed in Poromaster software v7.01. From the data obtained, the following were also determined:

1. P_c_—total porosity in [%];
2. V_tot_.—total pore volume in [cm^3^/g];
3. W—volume shares of the dominant pore populations, determined from the population pore size distribution curves in [cm^3^/g];
4. S—total pore surface in [m^2^/g];
5. A—average pore size in [nm]

### 2.6. Statistics

The results obtained in the tables and graphs are the arithmetic mean of the values from six measurements. The distribution of results did not exceed 0.1%.

## 3. Results

The results for the moisture parameters are presented in Table 3.

**Figure 1:**
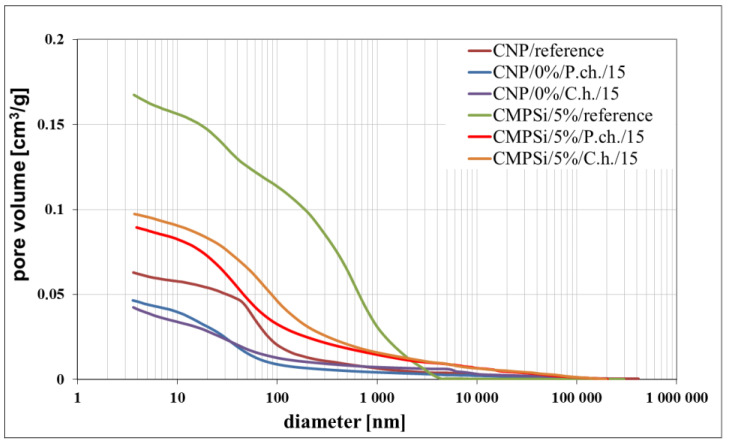
Cumulative curves for pore volume distribution in CNP and CMPSi mortars as a function of their diameters in the studied range of pore size for fungal contamination: *Penicillium chrysogenum* and *Cladosporium herbarum*.

Figure 1 presents a cumulative pore volume distribution curve for CNP and CMPSi mortars which were uncontaminated and contaminated with filamentous fungi. 

Table 4 shows the results from the porosimetry, i.e., the volume contribution of each pore range, total pore volume, total porosity, total pore surface area and average pore size. A simplified breakdown of the pores was presented in Section 1, and this was also used in this study but with one change which relates to capillary pores and macropores. The change was made after analysing the pore size distribution diagrams. It was noticed that, up to a size of 1000 nm, the pore volume changes noticeably, while above 5000 nm these changes are insignificant. Therefore, we decided to extend the range for capillary pores and reduce that for mesopores. For this reason, the following pore ranges were used in this study: >10 nm, 10 to 100 nm, 100 nm to 5000 nm and <5000 nm.

**Figure 2:**
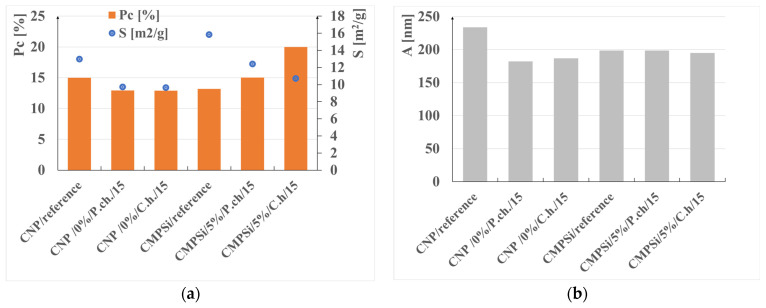
Graphs of the dependence of CNP and CMPSi materials—reference and contaminated with *Penicillium chrysogenum* and *Cladosporium herbarum* fungi—on (**a**) porosity (P) and total pore area (S); (**b**) average pore size (A).

Figure 2 shows the relationship between CNP and CMPSi materials—reference materials and those contaminated with *Penicillium chrysogenum* and *Cladosporium herbarum* fungi—on porosity, total pore area and average pore size.

**Figure 3:**
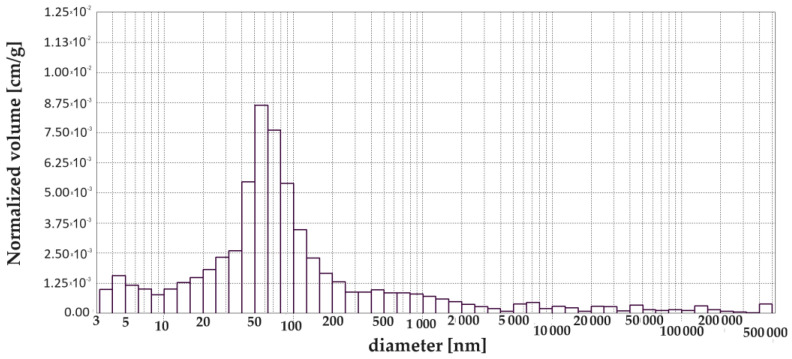
Dependence (histogram) of the pore volume in the CNP mortar on the pore diameter in the tested pore size range.

**Figure 4:**
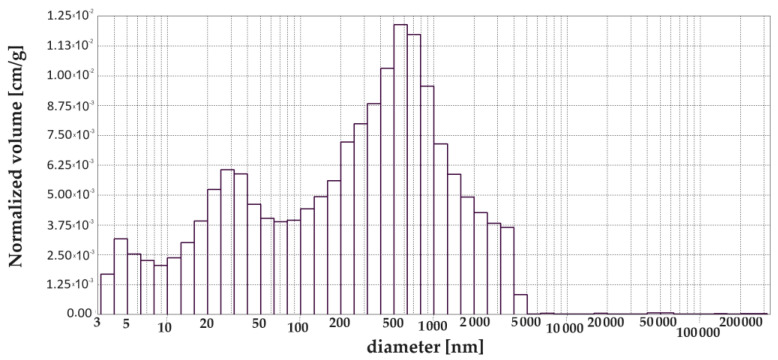
Dependence (histogram) of the pore volume in the CMPSi mortar on the pore diameter in the tested pore size range.

**Figure 5:**
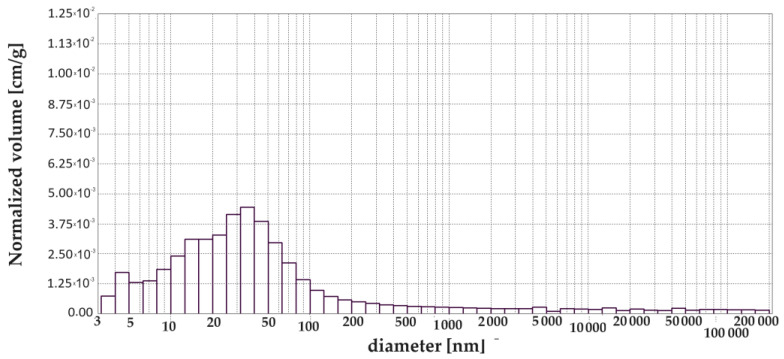
Graph (histogram) of pore volume in CNP mortar contaminated with *Penicillium chrysogenum* depending on the diameter in the tested pore size range (exposure time 15 months).

**Figure 6:**
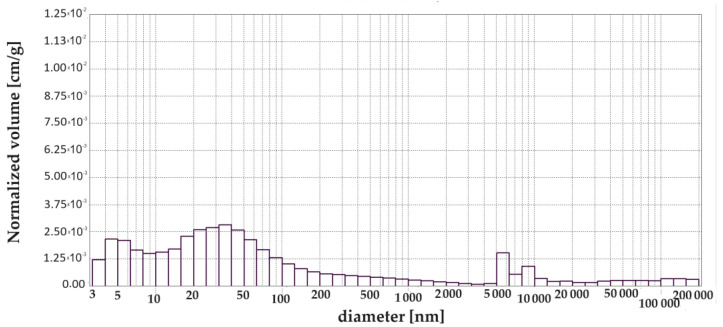
Graph (histogram) of pore volume in CNP mortar contaminated with *Cladosporium herbarum* depending on the diameter in the tested pore size range (exposure time 15 months).

**Figure 7:**
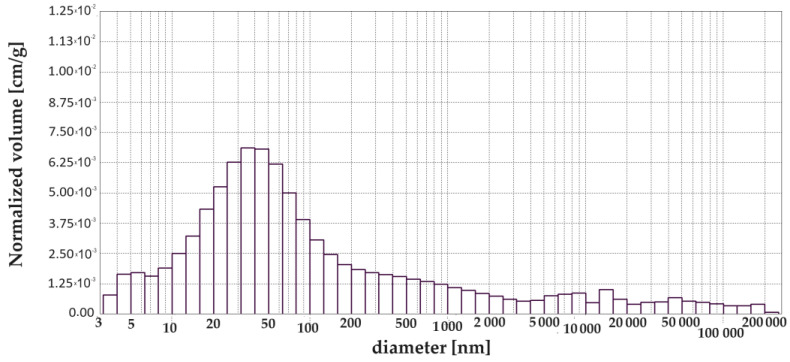
Graph (histogram) of pore volume in CMPSi mortar contaminated with *Penicillium chrysogenum* depending on the diameter in the tested pore size range (exposure time 15 months).

**Figure 8:**
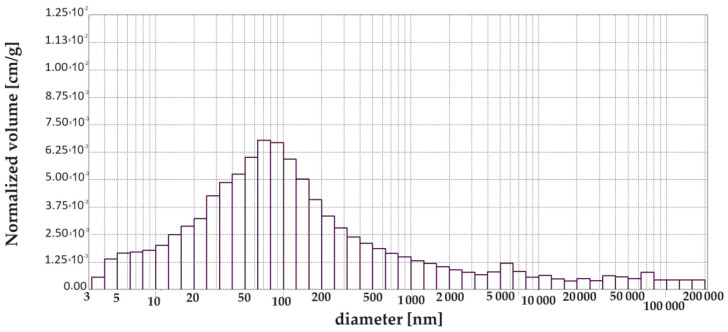
Graph (histogram) of pore volume in CMPSi mortar contaminated with *Cladosporium herbarum* depending on the diameter in the tested pore size range (exposure time 15 months).

Figure 3, Figure 4, Figure 5, Figure 6, Figure 7 and Figure 8 show histograms showing the pore volumes in uncontaminated and contaminated CNP and CMPSi mortars.

## 4. Discussion

This article focuses on the study of moisture content and its adsorption due to the secretion of water by filamentous fungi. In addition to this, they are also able to absorb moisture and accumulate it for their vital needs. Consequently, water will be found both on the surface of materials and inside them.

The influence of mould increased the moisture content of the material after a 15-month exposure period. The highest moisture content was found for material doped with polysiloxane and contaminated with *Penicillium*, but the lowest moisture content value for Cladosporium contamination was also noted for this material. In all contaminated materials, the mass moisture content increased compared to uncontaminated materials. Compared to the standard mortar, the mass moisture content increased by more than 100%. The mass moisture after 15 months of contamination with filamentous fungi significantly exceeds the absorbability for material without CNP admixture and with CMPSi admixture contaminated with *Penicillium chrysogenum*. This exceedance is also illustrated by the degree of moisture penetration. Only in the case of *Cladosporium* contamination did the degree of moisture penetration not exceed 100%. Comparing the unmixed materials with 5% admixture, it can be seen that CNP is the more permeable of the reference materials. However, after 15 months of exposure, the material with 5% admixture has the highest absorption for both *Penicillium* and *Cladosporium* contamination. Mortar wetting is usually a gradual process. It can also vary over time. It depends on the level of contamination and the amount of moisture produced by the biological environment. Moisture content and fungi content do not always correlate well with building materials [21]. Moisture can be caused by biofilm, which is produced by microorganisms. Water can accumulate in the biofilm to protect fungal life functions and alter climatic conditions.

Table 3 additionally contains two parameters related to the moisture content of the materials: the degree of water penetration and the degree of water saturation. In publication [22], a first attempt was made to adapt these parameters to cementitious materials contaminated with microbial environments. The first parameter is specific to the moisture content of ceramic walls. The second, on the other hand, depends on the volumetric absorbability and porosity of the sample. This means that it depends on the amount of water in the volume of the material and takes into account possible changes in the pore structure. 

The change in the degree of moisture penetration and the change in moisture content have the same tendency during the period of exposure to microorganisms. The degree of moisture penetration is indicated by the number of pores filled with water expressed as a percentage. Therefore, it indicates what percentage of the available pore volume is filled with water. For most materials exposed to the biological environment, the moisture penetration rate exceeds 100%. Only in the case of Cladosporium contamination did the degree of moisture penetration not exceed 100%. It is likely that the presence of a biofilm on the surface triggers this change.

Pore ranges were determined from the cumulative pore volume distribution curves obtained from the mercury porosimeter (MIP) test. The results for the volume contribution of the predominant pore populations are summarised in Table 4. The pores with specific ranges were largely involved in corrosion due to filamentous fungi. In the literature describing the aggression of different chemical environments, there is a discrepancy between the classification of pores and their nomenclature [4]. Therefore, a simplified classification was adopted in this study. Analysis of the charts allowed us to select the most important pores, which cover the ranges: >10, 10–100, 100–5000 nm. Therefore, a simplified pore size distribution has been used. 

The internal structure of the materials was analysed to determine pore type, pore volume and total porosity, which is presented in Table 4 and Figure 2a,b. The most important pore ranges were 10–100 nm for each material with and without admixture. An additional range of 100–500 nm was significant for the material with 5% polysiloxane admixture. 

The porosity of the cementitious materials varies from about 13 to almost 20%. The results in Figure 4 show that, in the case of CNP, contamination with both *Penicillium chrysogenum* and *Cladosporium herbarum* slightly sealed the structure after a 15-month exposure period. However, in the case of adding 5% admixture to CEM I, there was an increase in porosity after contamination with *Penicillium chrysogenum* as well as *Cladosporium herbarum*. Thus, the admixture unsealed the structure relative to the CEM I mortar. The values of the total pore area behave similarly to the porosity values, which decrease for mould-contaminated materials. However, this only happens for CEM I mortars. However, for CMPSi mortars it behaves completely differently. With the highest porosity, the value of the total pore area is the lowest and vice versa. In contrast, another parameter characterising the internal structure, i.e., the average pore size (results presented in Figure 3), is lower for CNPs exposed to the biological environment. For CMPSi, on the other hand, these values are virtually the same. This is the first time that total pore area and average pore size have been introduced into the biodeterioration analysis of CEM I and polymer-modified CEM I mortars, so it is difficult to determine whether these parameters will be useful for the analysis of the structure and changes resulting from the microbial environment.

With the graphical representation of the internal structure by means of the differential curves and the cumulative pore distribution shown in Figure 2 and the data in Table 4, it is possible to analyse the pore distribution and size. The pore volumes and volume shares of the predominant pore populations are shown in Table 4 and in Figure 3, Figure 4, Figure 5, Figure 6, Figure 7 and Figure 8 showing histograms of pore volumes. The histogram plots are intended to better illustrate the behaviour of the individual pore sizes of uncontaminated and filamentous fungus-contaminated mortars. Analysing the volume contribution of materials unmodified with admixtures, it can be seen that the total number of pores decreases when the environment is contaminated with both fungal species, with a slight increase in the number of small pores (less than 10 nm). The reason for the slight increase in small pores with a decrease in the total pore size may be that the larger pores are filled with metabolic products or fragments of shreds. When this happens, some of the large pores are completely closed and some are slightly opened (up to the size of the small pores). Products that may have filled the pores are, for example, calcium carbonate as a product of biogenic carbon dioxide and calcium hydroxide or the CSH phase. In the case of cement–polymer materials with an admixture of 5%, the total number of pores decreases:

- In the 10–100 nm range, the proportion of pores increases compared to the reference cement material.
- In the 100–500 nm range, the proportion of pores decreases compared to the reference material.
- New pores are formed in the range above 5000 nm.

When comparing cement–polymer composites with a 5% admixture to materials without doping, it is clear that the structure is less tight. This can be seen in both the proportion of pores and their total number.

The presence of filamentous fungi indicates that biodeterioration is occurring on the materials. This is a deterioration of the properties of technical materials caused by the activity of microorganisms, i.e., their growth and release of their metabolic products into the environment. The mechanism that is usually observed on mineral engineering materials is chemical dissimilatory biodeterioration, which occurs when microbial metabolites damage the material, causing chemical corrosion phenomena, pigmentation or the release of toxic metabolites into the material [22]. Moulds can secrete various metabolic products, i.e., volatile organic compounds, mycotoxins, pigments, acidic metabolic products and water. The first two affect human health, while construction/engineering materials are most affected by the last two substances. Given that most engineering materials are porous, water released by microorganisms can accumulate in them. The more porous the structure of the material (the more open pores there are), the more water it can absorb. In the case of cement mortar, the volumetric moisture content ranges from 1.8 to 5.6% [23]. The more open pores of a size that allows water to penetrate, the greater its absorbability, i.e., the more water will penetrate the material. If the material is wet, then better conditions will be created for the growth of microorganisms on such a material. On the other hand, if the material contains closed pores, the water does not affect the material as much, because it does not enter these closed pores.

Both cementitious materials were found to be susceptible to mould. However, CEM I cementitious materials without admixtures were found to be slightly less susceptible to mould after 15 months of exposure to mould than CEM I with the addition of 5% polysiloxane admixture. The polysiloxane admixture, due to its other functions in the concrete, not only increased the porosity of the mortar, but caused the pore distribution to be unfavourable compared to the mortar not modified with the polymer. Consequently, the pathway for microbial growth was facilitated. The structures of the unmodified mortars contaminated with fungi of both types sealed slightly, but the pore distribution also changed. Compared to the data in publication [24], there was a slight sealing of the structure as a result of the biological environment, but this was due to corrosion products. In contrast, CEM I mortars with a 5% polysiloxane latex admixture continued to unseal when compared to the data in publication [16].

## 5. Conclusions

The article presents the impact of the moulds *Penicillium chrysogenum* and *Cladosporium herbarum* over a period of 15 months on materials made of CEM I, one of which was unmodified and the other was a cement–polymer composite with a 5% admixture of polysiloxane latex. In particular, the internal structure was analysed, which included the size and distribution of pores and the moisture parameters of these materials.

The key conclusions from this study can be summarized as follows:

- The highest increase in humidity occurred in mortars modified with polysiloxane and contaminated with *Penicillium chrysogenum*, while the smallest increase occurred in the same material but contaminated with *Cladosporium herbarum*;
- The largest changes in the share of pores occurred in the range from 10 to 100 nm and from 100 to 500 nm, this applies to both materials and both corrosion environments;
- As a result of the action of microorganisms, changes in the tightness of cement materials, including those containing organic modifiers, occur for 15 months;
- The mortar not modified with polymer was sealed under the influence of mould fungi. However, cement–polymer mortars were leaking;
- For the first time, the total pore area and average pore size were introduced for the analysis of biodeterioration of CEM I mortars and polymer-modified CEM I mortars, therefore it is difficult to determine whether these parameters will be useful for analysing the structural changes resulting from the microbiological environment. Therefore, these changes should be analysed in all periods of exposure to filamentous fungi and for various materials.

## Figures and Tables

**Table 1:** Chemical composition of the cement used.

Content [%mass.]	CaO	SiO_2_	Al_2_O_3_	Fe_2_O_3_	Sulphates SO_3_	MgO	K_2_O	Na_2_O	Cl
CEM I 42.5	63.05	18.74	4.90	3.17	2.8	1.32	0.89	0.13	0.01

**Table 2:** Composition of the cement–polymer mortars tested.

Designation	Content [g]			
	Cement	Sand	Water	Polymer Modifier
CNP	100	300	50	-
CMPSi (polysiloxane latex)	100	300	47.5	5

**Table 3:** Results of moisture parameters for cement–polymer composites.

**Parameter**		**Material**	**CNP**			**CMPSi**		
		**Time [Month]**	**0**	**15**	**15**	**0**	**15**	**15**
	**Environmental**			**P.ch.**	**C.h.**	**P.ch.**		**C.h.**
mass moisture [%mass.]			2.28	6.72	6.55	1.87	7.98	3.70
absorbability [%mass.]			6.75	6.46	6.13	6.07	7.35	6.57
degree of moisture permeation [%]			33.82	104.02	106.85	30.78	108.57	56.32
degree of saturation with water			0.450	0.499	0.475	0.304	0.534	0.424

**Table 4:** Volume shares of dominant pore populations in the tested materials.

Mortar/Quantity/ Contamination/Time	W [cm^3^/g]						V_tot_
	10	10–100	100–5000	<5000	10–5000	Total	[cm^3^/g]
CNP/reference	0.005	0.038	0.016	0.004	0.059	0.063	0.063
CNP/0%/P.ch./15	0.007	0.031	0.006	0.003	0.044	0.046	0.046
CNP/0%/C.h./15	0.009	0.022	0.006	0.006	0.037	0.043	0.042
CMPSi/reference	0.011	0.043	0.113	0.000	0.167	0.167	0.167
CMPSi/5%/P.ch/15	0.007	0.050	0.023	0.009	0.081	0.089	0.089
CMPSi/5%/C.h/15	0.007	0.050	0.031	0.009	0.089	0.098	0.097

## Data Availability

Data are contained within the article.
